# A steroid-resistant nephrotic syndrome in an infant resulting from a consanguineous marriage with *COQ2* and *ARSB* gene mutations: a case report

**DOI:** 10.1186/s12881-019-0898-4

**Published:** 2019-10-28

**Authors:** Xia Wu, Wenhong Wang, Yan Liu, Wenyu Chen, Linsheng Zhao

**Affiliations:** 1 Department of Nephrology, Tianjin Children’s Hospital, 238 Longyan Road, Beichen District, Tianjin, China; 2 Department of Pathology, Tianjin Children’s Hospital, 238 Longyan Road, Beichen District, Tianjin, China

**Keywords:** *COQ2* gene, *ARSB* gene, Steroid-resistant nephrotic syndrome, Child

## Abstract

**Background:**

Treatment of steroid-resistant nephrotic syndrome (SRNS) remains a challenge for paediatricians. SRNS accounts for 10~20% of childhood cases of nephrotic syndrome (NS). Individuals with SRNS overwhelmingly progress to chronic kidney disease (CKD) and end-stage kidney disease (ESRD). Genetic research is of great significance for diagnosis and treatment. More than 39 recessive or dominant genes have been found to cause human SRNS, including *COQ2*. *COQ2* gene mutations not only cause primary coenzyme Q10 deficiency but also cause SRNS without extrarenal manifestations. The concept of *COQ2* nephropathy has been proposed for a long time. Mutations in the *COQ2* gene have rarely been reported. Worldwide, only 5 cases involving 4 families have been reported.

**Case presentation:**

We present the case of a 6-month-old girl with steroid-resistant glomerulopathy due to a *COQ2* defect with no additional systemic symptoms. The patient was identified as a homozygote for the c.832 T > C (p. Cys278Arg) missense mutation and a single base homozygous mutation in *ARSB* gene in c.1213 + 1G > A. The father and mother were heterozygous mutation carriers in both *COQ2* and *ARSB*, and her healthy sister was only a heterozygous mutation carrier in *COQ2*. In this case, hormone therapy was ineffective, and progressive deterioration of renal function occurred within 1 week after onset, leading to acute renal failure and eventual death.

**Conclusions:**

We reported a consanguinity married family which had *COQ2* and *ARSB* dual mutant. Kidney diseases caused by *COQ2* gene mutations can manifest as SRNS, with poor prognosis. The C. 832 T > c (p.csc 278arg) is a new mutation site. Genetic assessment for children with steroid-resistant nephrotic syndrome, especially in infancy, is very important. Families with a clear family history should receive genetic counselling and prenatal examinations, and children without a family phenotype should also receive genetic screening as early as possible.

## Background

Mutations in the *COQ2* gene can lead to coenzyme Q10 (COQ10) deficiency, a type of mitochondrial encephalomyopathy and kidney disease, an inherited mitochondrial disease that mainly affects the kidneys. The severity of kidney disease varies and is not necessarily associated with neurological symptoms. Steroid-resistant nephrotic syndrome (SRNS) accounts for 10~20% of childhood cases of nephrotic syndrome (NS) [1, 2]. Individuals with SRNS overwhelmingly progress to chronic kidney disease (CKD) and end-stage kidney disease (ESRD). More than 39 recessive or dominant genes have been found to cause human SRNS, including *COQ2* mutations. Patients with recessively inherited mutations manifest with SRNS in childhood [3]. Overall, whole-exome sequencing of 300 SRNS patients showed that 23 known single gene aetiological mutations of SRNS were detected, among which only one gene mutation related to *COQ2* was detected [4]. At present, the reported gene mutation types include compound heterozygotes for c.590G > A (p.arg197his) and c. 683a > G(p.sn228ser), c. 683a > G(p.sn228ser) and c. 683a > G(p.sn228ser), and homozygotes for 701delT, c.437G > A(p.ser146asn) and c.890A > G(p.tyr297cys). Mucopolysaccharidosis VI (MPS VI) is a very rare autosomal recessive disorder caused by mutations in the *ARSB* gene. To date, 340 mutations have been described, most of which are single base homozygous mutations.

## Case presentation

**Fig. 1:**
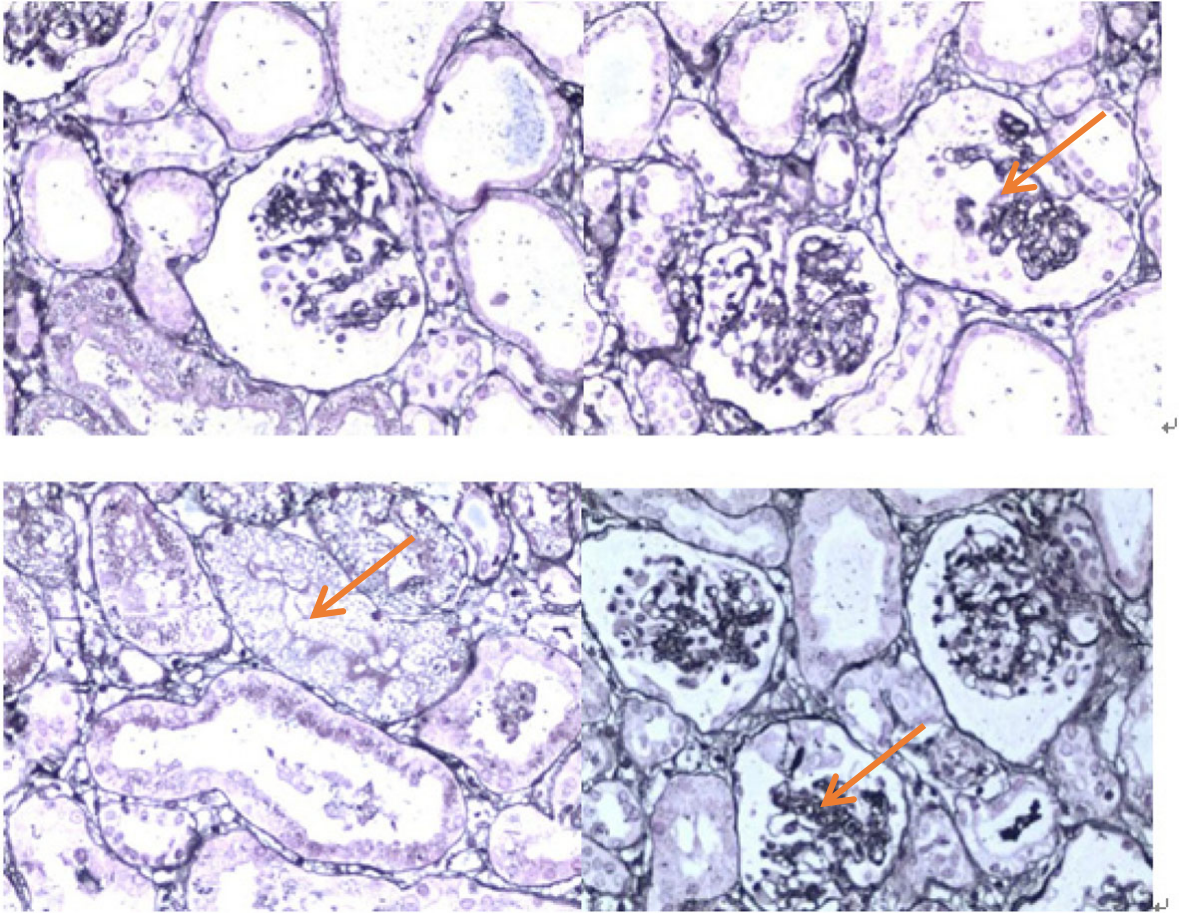
Renal histology and ultrastructural findings in light microscopy. It showed 48 glomerular, 4/48 were immature glomerular; 17/48 had glomerular mesangial cells and matrix hyperplasia with different levels of insert appearance (glomerular collapse, did not see open capillary lumens); 15/48 had glomerular balloon expansion, the expansion of multiple focal proximal convoluted tubules, renal tubular epithelial lesions sliced and interstitial oedema; and small arteries did not show obvious pathological changes. Immunohistochemical tests results of 4 glomerular sections: IgG(−), IgA(−), IgM(−), C3(−), C1q(−), and Fn(−) (haematoxylin and eosin stain). Magnifications: × 200

**Fig. 2:**
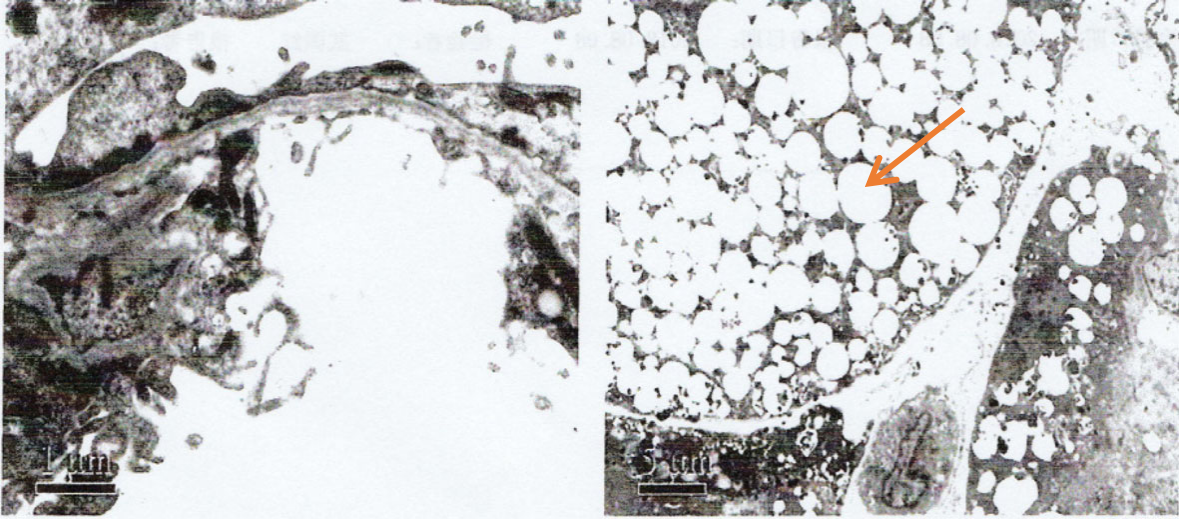
Renal histology and ultrastructural findings in electron microscopy. Renal histology and ultrastructural findings in the electron microscopy image showed no obvious changes in the glomerular basement membrane, extensive fusion of epithelial cells, vacuolar degeneration of podocytes, and no deposition of electronic dense matter. The renal tubular epithelium showed obvious vacuolar degeneration with varying sizes. There were flocculent/granular electronic dense substances in the vacuole, some with core particles, and increased lysosomes, partial shedding and partial atrophy of microvilli, and a small number of mononuclear lymphocytes infiltrating into the renal interstitium, showing vacuolar structure (hexaammonium silver staining). Magnifications: × 6700

**Fig. 3:**
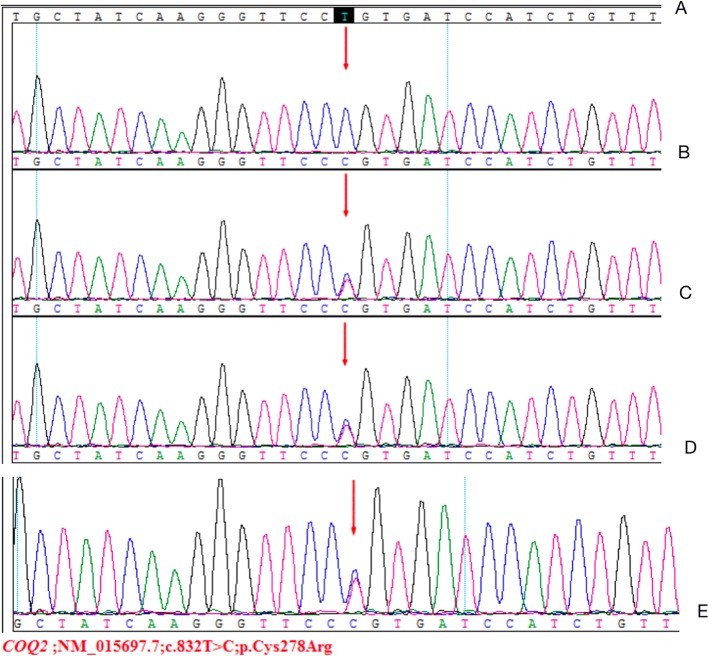
Analysis of *COQ2* gene mutation in the family with affected children: **a** Normal control sequence. **b** Homozygous mutation of this child for c.832 T > C. **c** Heterozygous mutation of her father for c.832 T > Cr. **d** Heterozygous mutation of her mother for c.832 T > C. **e** Heterozygous mutation of her sister for c.832 T > C

**Fig. 4:**
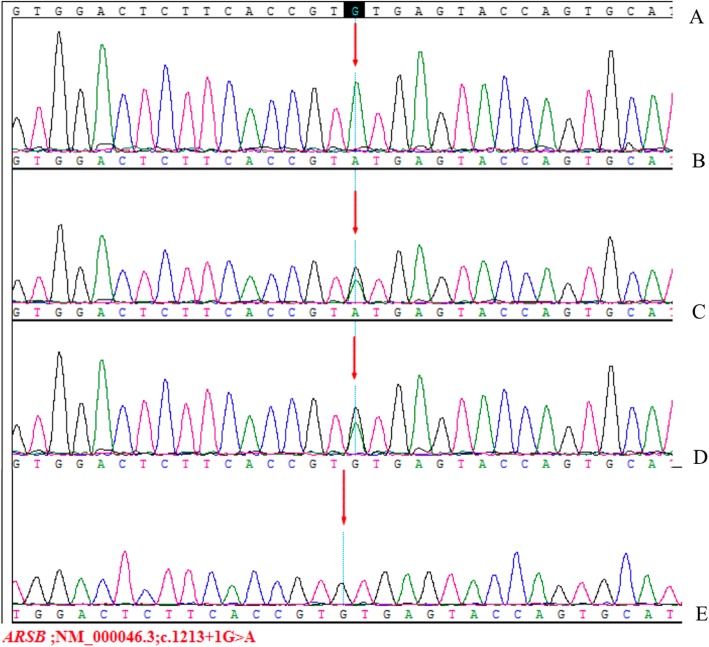
Analysis of *ARSB* gene mutation in the family with affected children: **f** Normal control sequence. **g** Single base homozygous mutation of this child for c.1213 + 1G > A. **h** Heterozygous mutation of her father for c.1213 + 1G > A. **i** Heterozygous mutation of her mother for c.1213 + 1G > A. **j** Her sister did not have this gene mutation

**Fig. 5:**
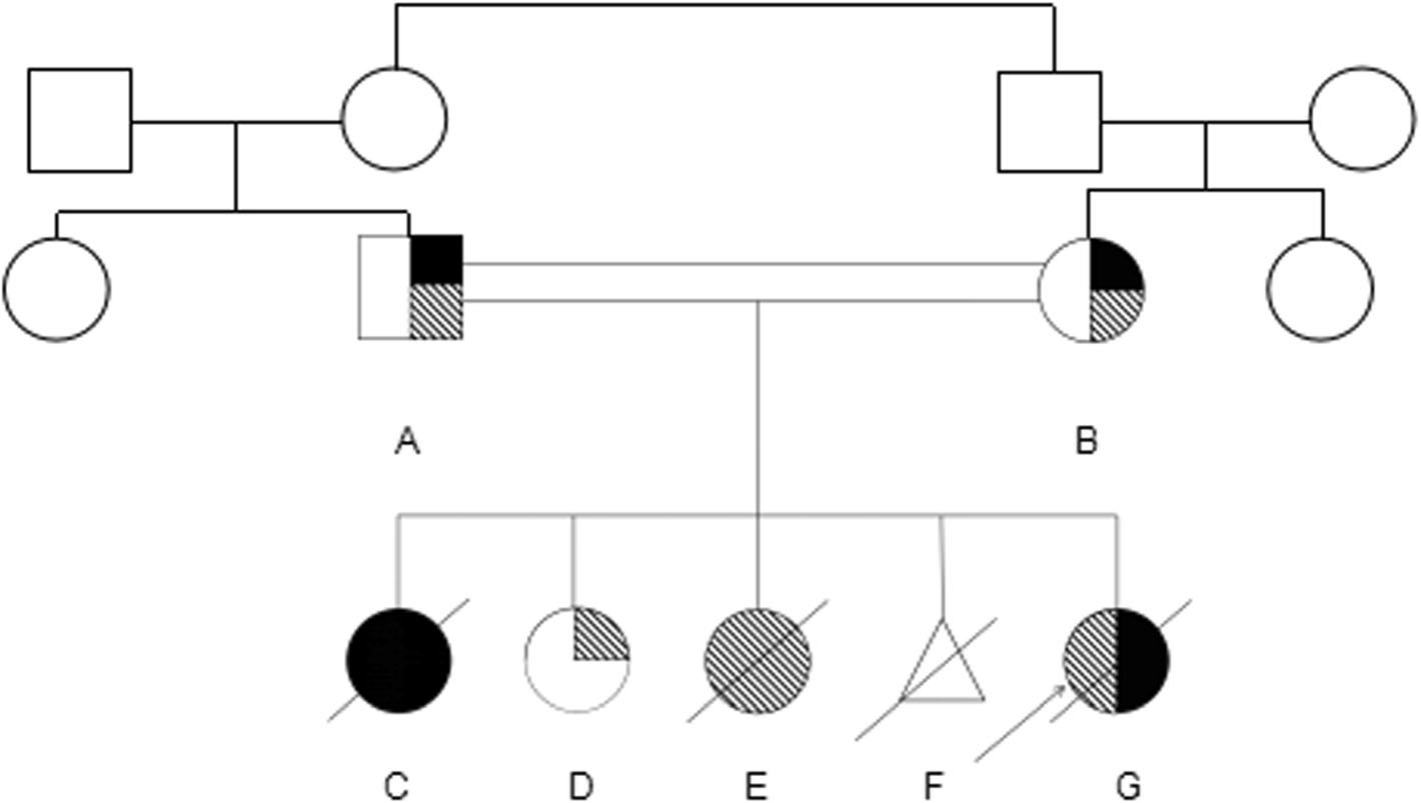
Family tree **a**: The father carried the heterozygous mutation gene of *COQ2* and *ARSB*, and the phenotype was normal. **b**: The mother carried a heterozygous mutation gene of *COQ2* and *ARSB*, and the phenotype was normal. **c**: The first daughter was diagnosed with mucopolysaccharide storage disease, deceased. **d**: The second daughter is a heterozygous mutation carrier of the *COQ2* gene, with a normal phenotype. **e**: The third daughter was diagnosed with kidney syndrome, deceased. **f**: The fourth child was aborted after 5 months of gestation. **g**: The fifth daughter carried homozygous mutations of the *COQ2* and *ARSB* genes and died from nephrotic syndrome

A 6-month-old female infant was admitted to the nephrology department of Tianjin Children’s Hospital, China, on July 21, 2018. Physical examination revealed anasarca, cardiopulmonary examination showed no obvious abnormality or abdominal bulge, liver and spleen were not enlarged, and epileptic seizure, ataxia or facial deformity were present. Serological examination presented with massive proteinuria (5.657 g/d), hypoalbuminemia (serum albumin 18 g/L), hypercholesterolemia (serum total cholesterol 8.23 mmol/L), and lactic acidosis (4.32 mmol/L). The child’s parents are cousins and sisters, both of whom are Han, and they are in good health. Their first child was a girl who was diagnosed with mucopolysaccharides at the age of 6 and died at 11 years old. The second child was a 12-year-old healthy girl. The third girl was diagnosed with nephrotic syndrome at 8 months old; treatment was halted, and the child died. The fourth child was aborted at 5 months. The fifth is the child in this report. The mother had no pregnancy complications and was not on any special medication. The child was treated with methylprednisolone. There was no remission trend in proteinuria, showing hormone resistance. The patient rapidly developed acute renal failure within 1 week and underwent peritoneal dialysis. Renal pathological and gene examinations were performed over time. Light microscopy images showed 48 glomeruli, 4/48 as immature glomeruli, 17/48 of glomerular mesangial cells and matrix hyperplasia with different levels of insert appearance (glomerular collapse, did not see open capillary lumens), 15/48 of the glomerular balloon expansion, the expansion of multiple focal proximal convoluted tubules, sliced renal tubular epithelial lesions and interstitial oedema, and the small arteries did not show obvious pathological changes. Immunohistochemical testing of 4 glomeruli results include IgG(−), IgA(−), IgM(−), C3(−), C1q(−), and Fn(−) (Fig. 1). Electron microscopy showed no obvious changes in the glomerular basement membrane, extensive fusion of epithelial cells, vacuolar degeneration of podocytes and no deposition of electronic dense matter. The renal tubular epithelium showed obvious vacuolar degeneration with varying sizes. There were flocculent/granular electronic dense substances in the vacuoles, some with core particles, increased lysosomes, partial shedding and partial atrophy of microvilli, and a small number of mononuclear lymphocytes infiltrating into the renal interstitium, showing vacuolar structure (Fig. 2). The *COQ2* gene has a homozygous mutation for c.832 T > C (p.cys278arg), and both her parents and sister were heterozygous (Fig. 3). In addition, another likely pathogenic mutation was detected in the patient’s *ARSB* gene, which was c.1213 + 1G > A, which was a single base homozygous (Hom) mutation. Both parents were carriers of a heterozygous mutation in the *ARSB* gene, which her sister did not have (Fig. 4). SIFT software was used to predict the function of c.832 T > C (p.cys278arg) protein, and the results were harmful (Fig. 5). After several days of peritoneal dialysis, her parents eventually gave up treatment, and the child died 2 weeks later.

## Discussion

*COQ2* (OMIM609825) is located at 4q21.22-q21.23 and has 17 exons. As early as 1975 and 1990, scholars observed that mutations in the gene encoding mitochondrial coenzyme Q10 synthase could lead to a decrease in COQ10 (MIM607426) content and affect the electron transport of the respiratory chain. In 1992, Matthew N. Ashby, an American scholar, showed that *COQ2* could encode polypentene hydroxybenzoate transferase, which is a very important enzyme in the synthesis of COQ10. It can clear oxygen free radicals and reduce oxidative stress reactions. COQ10 deficiency is an autosomal recessive genetic disease that is related to four main clinical phenotypes, including prominent nephropathy and encephalopathy, which are typical infant multi-system diseases. Scholars reported a 33-month-old boy with infant brain myopathy, kidney disease and COQ10 deficiency, and a homozygous missense mutation was found in *COQ2* genes after genetic testing. For the first time, *COQ2* gene coding mutations can lead to primary COQ10 deficiency. Recent studies have found that mitochondrial nephropathy can occur independently without the manifestations of mitochondrial encephalomyopathy, and hormone therapy and immunosuppressive therapy are ineffective. In 2007, scholars reported two cases of patients with early onset of glomerular lesions, and both cases have found *COQ2* gene mutations; the study puts forward the concept of “*COQ2* kidney disease”. It was described as a new genetic mitochondrial disease with primary kidney involvement and is characterized by variable renal lesions and the proliferation of mitochondria with an aberration in glomerular cells, and the clinical manifestations may be polymorphic. Additionally, neuromuscular symptoms may complicate the course of disease. The most severe forms are devastating multi-system disorders, including glomerular dysfunction and lactic acidaemia [5]. The gene defects associated with human hereditary nephrotic syndrome, especially the *COQ2* gene, can cause ultrastructural abnormalities of the hiatus membrane channel, hole membrane channel collapse, mitochondrial malformation increase, elevated levels of autophagy and mitosis in renal cells, increased reactive oxygen species, and increased susceptibility to oxidative stress [6]. Lipid peroxidation and mitochondrial dysfunction are associated with congenital nephrotic syndrome and glomerular proteinuria. British scholars have conducted gene testing on 24 children with hormone-resistant nephrotic syndrome and found that 1 child had a *COQ2* mutation. There were no clinical manifestations outside the kidney, and the condition eventually developed into renal failure [7]. For coenzyme q10 deficiency, homozygous mutations are often a sign of serious disease [8].

Of the six cases reported in the current literature [7, 9] (Table 1), 4 cases were homozygous mutations. Homozygous mutations in the *COQ2* gene usually lead to early onset SRNS. The parents of the four cases were in consanguineous marriages. Two patients developed acute renal failure (ARF), received peritoneal dialysis, and died at 6 months. If COQ10 deficiency has been identified clinically, but pathogenicity variation is unknown, measurement of COQ10 level in skin fibroblasts can be considered to assess whether it is high risk [10]. COQ10 supplementation can significantly improve the prognosis but requires early treatment [11]. Di Giovanni S reported a 33-month-old boy, and oral administration of COQ10 improved the nervous system but did not improve renal function. More research is needed in the treatment of kidney diseases.

**Table 1:** Clinical, pathological, biochemical, and genetic features of reported patients

Parameter	Patient					
	1	2	3	4	5	6
Reference	This report	Refs [9].	Refs [9].	Refs [9].	Refs [9].	Refs [7].
Family history						
Consanguinity	Yes	No	Yes	Yes	Yes	No
Other siblings	1 healthy sister, 1 dead sister (died at 11 years old),1 dead sister (died at 8 mons old)	1 healthy brother	1 healthy sister, 1 dead sister (at 2 d of life)	Patient 5	Patient 4	No
Gender	Female	Male	Male	Male	Female	Female
Renal involvement						
Renal symptoms	SRNS/AFR	SRNS	ARF	SRNS	NS	SRNS
Age of onset	6 mo	18 mo	Birth	11 mo	12 mo	18 mo
Renal pathology	Extensive fusion of epithelial pedocytes, degeneration of podocyte vacuoles, tubular-interstitial lesion	Collapsing GN	Crescentic GN	FSGS	FSGS	Not mentioned
Treatment	Meprednisone	ACEI indomethacin(18 mo) CoQ10 (21 mo)	Prednisone	Prednisone	Prednisone	COQ10
Out come	Death(6 mo)	ESRD(20 mo)	Death(6 mo)	ESRD(18 mo)	Normal renal function	ESRD(30 mo)
Extrarenal involvement	None	None	Epileptic encephalopathy, hypotonia	Epilepti c encephalopathy, optic nerve atrophy	None	None
*COQ2* mutations	Homozygous c.832 T > C (p.cys278Arg)	Combined heterozygous c.590G > A (p.Arg197His) c.683A > G (p.Asn228Ser)	Homozygous c.437G > A (p.Ser146Asn)	Homozygous c.890A > G (p.Tyr297Cys)	Homozygous c.890A > G (p.Tyr297Cys)	Compound heterozygousc.683A > G (p.Asn228Ser) c.701delT

*AFR* acute renal failure, *NS* nephrotic syndrome, *GN* glomerulonephritis, *FSGS* focal nodular glomerulosclerosis, *ACEI* angiotensin converting enzyme inhibitors, *ESRD* end-stage kidney disease

SRNS steroid resistant nephrotic syndrome

The *ARSB* gene is related to mucopolysaccharidosis type VI (MIM no. 253200). *ARSB* is located on chromosome 5q14.1 and has ubiquitous expression in the kidney, but no renal disease has been reported. It clinically presents multiple systemic symptoms and chronic progression, mainly involving the bone and cardiopulmonary system, cornea, skin, liver, spleen, brain and nerve. Severe MPS VI usually begins before the age of 3 years [12]*.* Naglazyme (galsulfase) was approved as an enzyme replacement therapy (ERT) for MPS VI in May 2005 and was considered a safe and effective treatment. With the advent of ERT, early application can benefit patients more, and early recognition and accurate diagnosis of this rare lysosomal storage disease are mandatory for a better and successful outcome [10]. Supportive care and bone marrow transplantation were the only treatments available for MPS VI patients. Therefore, early detection and diagnosis with an immediate intervention are crucial for a better outcome.

There is no literature on COQ2 combined with ARSB gene mutations. In this case, the parents of the child were carriers of two pathogenic genes, COQ2 and ARSB, and both diseases were autosomal recessive inheritance. The c.832 T > C(p.cys278Arg) of COQ2 is a newly discovered locus. The mutation type was a single base homozygous mutation (Hom), and this gene locus has not been publicly reported. The child developed renal failure and eventually died, suggesting that the poor prognosis was related to the carrying of homozygous mutant genes. The parents of this child had given birth to a girl with a definite diagnosis of MPS, so we can speculate that the ARSB gene carried by their parents is a pathogenic gene, and there is no related literature about the pathogenicity of this locus for the time being. In this case, there is no MPS VI phenotype, so it is not possible to determine whether kidney damage is related to this gene. At present, there is no literature on renal damage and pathological changes of MPS; thus, it is not clear whether the pathological changes of this case are caused by MPS. In addition, the renal pathology of this case did not find the mitochondrial proliferation manifestations reported in other literature on COQ2 gene mutation. However, it was only 10 d from the onset of this case to the renal pathological examination; the renal pathology likely had not changed due to the short period since onset. At the time of renal pathological examination, the child was in the stage of acute renal failure and needed renal replacement therapy. The pathological results showed that the renal tubular epithelial and interstitial oedema was relatively serious, which may be one of the reasons for the rapid deterioration of renal function.

## Conclusions

The c.832 T > C(p.cys278Arg) mutation in *COQ2* is a newly discovered locus. We believe that if the onset age is early, homozygous mutation and/or neuropathy are the risk factors for the severe cases resulting from the *COQ2* mutation. Consanguineous marriage is the cause of homozygous mutation. Genetic counselling should be carried out for whole family members, either prenatally or postnatally. Families with autosomal recessive diseases should avoid consanguineous marriages and inform their offspring of the risk of illness. Gene detection should be carried out as early as possible for foetal and normal phenotypes.

## Data Availability

All data generated or analysed during this study are included in this published article [and its supplementary information files].

## Abbreviations

*ARSB*: Aromatic sulfatase B
CKD: Chronic kidney disease
COQ10: Coenzyme Q10
ESRD: End-stage kidney disease
MPS VI: Mucopolysaccharidosis VI
NS: Nephrotic syndrome
SRNS: Steroid-resistant nephrotic syndrome
